# Risk of SARS-CoV-2 reinfection by vaccination status, predominant variant and time from prior infection: a cohort study, Reggio Emilia province, Italy, February 2020 to February 2022

**DOI:** 10.2807/1560-7917.ES.2023.28.13.2200494

**Published:** 2023-03-30

**Authors:** Massimo Vicentini, Francesco Venturelli, Pamela Mancuso, Eufemia Bisaccia, Alessandro Zerbini, Marco Massari, Andrea Cossarizza, Sara De Biasi, Patrizio Pezzotti, Emanuela Bedeschi, Paolo Giorgi Rossi, Reggio Emilia COVID-19 Working Group, Members of the Reggio Emilia COVID-19 Working group, Letizia Bartolini, Giorgia Bartolucci, Maria Chiara Bassi, Isabella Bisceglia, Maria Barbara Braghiroli, Valeria Cenacchi, Francesca Pia Lionetti, Nadia Montanari, Nicoletta Patrignani, Cinzia Perilli, Annamaria Pezzarossi, Francesca Roncaglia, Mara Santagiuliana

**Affiliations:** 1 Epidemiology Unit, Azienda USL-IRCCS of Reggio Emilia, Reggio Emilia, Italy; 2 Public Health Unit, Azienda USL-IRCCS di Reggio Emilia, Reggio Emilia, Italy; 3 Unit of Clinical Immunology, Allergy and Advanced Biotechnologies, Azienda Unità Sanitaria Locale-IRCCS di Reggio Emilia, Italy; 4 Infectious Disease Unit, Azienda USL-IRCCS di Reggio Emilia, Reggio Emilia, Italy; 5 Department of Medical and Surgical Sciences for Children and Adults, University of Modena and Reggio Emilia School of Medicine, Modena, Italy; 6 National Institute for Cardiovascular Research, Bologna, Italy; 7 Dipartimento Malattie Infettive, Istituto Superiore di Sanità, Rome, Italy; 8 The working group members are listed under Collaborators.

**Keywords:** Sars-CoV-2, Omicron, Reinfection, Immunity, Epidemiology

## Abstract

**Background:**

Understanding the epidemiology of reinfections is crucial for SARS-CoV-2 control over a long period.

**Aim:**

To evaluate the risk of SARS-CoV-2 reinfection by vaccination status, predominant variant and time after first infection.

**Methods:**

We conducted a cohort study including all residents in the Reggio Emilia province on 31 December 2019, followed up until 28 February 2022 for SARS-CoV-2 first infection and reinfection after 90 days. Cox models were used to compare risk of first infection vs reinfection, adjusting for age, sex, vaccine doses and comorbidities.

**Results:**

The cohort included 538,516 residents, 121,154 with first SARS-CoV-2 infections and 3,739 reinfections, most in the Omicron BA.1 period. In the pre-Omicron period, three doses of vaccine reduced risk of reinfection by 89% (95% CI: 87–90), prior infection reduced risk by 90% (95% CI: 88–91), while two doses and infection reduced risk by 98% (95% CI: 96–99). In the Omicron BA.1 period, protection estimates were 53% (95% CI: 52–55), 9% (95% CI: 4–14) and 76% (95% CI: 74–77). Before Omicron, protection from reinfection remained above 80% for up to 15 months; with Omicron BA.1, protection decreased from 71% (95% CI: 65–76) at 5 months to 21% (95% CI: 10–30) at 22 months from the first infection. Omicron BA.1 reinfections showed 48% (95% CI: 10–57) lower risk of severe disease than first infections.

**Conclusions:**

Natural immunity acquired with previous variants showed low protection against Omicron BA.1. Combined vaccination and natural immunity seems to be more protective against reinfection than either alone. Vaccination of people with prior infection reduced the risk of severe disease.

**Key public health message**

**What did you want to address in this study?**

The surge of Omicron variant of SARS-CoV-2 led to a rapid increase in reinfections, suggesting a reduced protection of immunity from earlier infections. We aimed to understand the protection against reinfection given by infection in the pre-Omicron and Omicron BA.1 periods of the pandemic and how it is related to vaccination history and severity of the first infection.

**What have we learnt from this study?**

In a highly vaccinated population, protection from a previous infection was strong against reinfections before the surge of the Omicron variant but only moderate against Omicron BA.1 reinfections. In this context, earlier infection was shown to be protective for nearly 2 years. Severe symptoms during the first infection, and hybrid immunity induced by vaccination and natural infection, provided a higher protection against Omicron BA.1 reinfections compared to either alone. We also found a halved risk of severe COVID-19 in Omicron BA.1 reinfections, compared with primary infections while only a slight, if any, reduction was found in the pre-Omicron period.

**What are the implications of your findings for public health?**

Evidence of a much higher protection of hybrid immunity against reinfection, compared with vaccination or prior infection alone, as well as the effect of disease severity on protection against Omicron reinfection generate hypotheses to be explored in future biological and epidemiological studies.

## Introduction

When the coronavirus disease (COVID-19) pandemic began in early 2020, the absence of pre-existing protective immunity to the severe acute respiratory syndrome coronavirus 2 (SARS-CoV-2) caused the rapid spread of the infection across the world. By 28 February 2023, 675,178,713 COVID-19 cases had been reported worldwide, with over 6.8 million deaths [1]. Given the high prevalence of SARS-CoV-2 infections globally, the incidence of reinfections – known to occur since as early as June 2020 [2,3] – has increased over time [4,5]. 

The occurrence of reinfections depends mainly on four driving forces: the increase in the number of people with a primary SARS-CoV-2 infection, the time elapsed since the first infection because of consequent waning of natural immunity, vaccine-induced immunity and the spread of different variants that can escape natural and vaccine-induced immunity. Despite knowledge gained on these factors [6-11], uncertainties remain on the long-term duration of natural immunity and how it interacts with vaccination (i.e. hybrid immunity) to decrease reinfection rates, particularly after the SARS-CoV-2 Omicron BA.1 (Phylogenetic Assignment of Named Global Outbreak (Pango) lineage designation B.1.1.529) variant became dominant in January 2022 [8]. Preliminary data from United Kingdom surveillance and a recent systematic review show lower protection of prior infections for infection by Omicron [12-14]. 

The Emilia-Romagna region was one of the first Italian regions impacted by the COVID-19 pandemic. By the end of February 2022, the Emilia-Romagna region had experienced four main COVID-19 waves, driven by the SARS-CoV-2 wild-type, followed by the Alpha (Pango lineage designation B.1.1.7), Delta (Pango lineage designation B.1.617.2) and Omicron BA.1 variants. On 28 February 2022, the cumulative SARS-CoV-2 detection rate in Emilia-Romagna was 26,715.73 per 100,000 residents and more than 90% of people older than 12 years received a full COVID-19 vaccination schedule (two doses) [15].

The aim of this cohort study based on routine COVID-19 surveillance data was to estimate the incidence of SARS-CoV-2 reinfections in all residents of Reggio Emilia, a province within the Emilia-Romagna region, since the start of the pandemic through 28 February 2022, according to vaccination status, predominant viral lineages, time since primary infection, characteristics of the patient and severity of the first disease.

## Methods

### Study design, setting and population data sources

We conducted a population-based cohort study based on COVID-19 surveillance data from the province of Reggio Emilia. This province has a population of over 538,000 inhabitants and is located in the Emilia-Romagna region, northern Italy. All individuals, regardless of age, who were residents of the Reggio Emilia province on 31 December 2019 and alive on 20 February 2020 were included.

Residency status, age and sex were retrieved by the Population Registry of the Local Heath Authority of Reggio Emilia.

Data on SARS-CoV-2 microbiologically diagnosed cases were routinely collected within the National COVID-19 surveillance registry (coordinated by the Italian National Institute of Health and implemented in each local health authority including the Local Health Authority of Reggio Emilia, providing healthcare services for all the Reggio Emilia province population [16]). The National COVID-19 surveillance registry collects data on SARS-CoV-2 cases confirmed by a positive test according to the current diagnostic and testing policy. Valid tests in Italy during the study period were reverse transcription PCR (RT-PCR) on nasal or nasopharyngeal swabs, third-generation antigenic tests (from January 2021) and other antigenic tests (from January 2022). Since 19 January 2022, in the Emilia-Romagna region, there was also the possibility of carrying out a rapid antigen self-test and uploading it to a web platform for case confirmation and notification to the National Health Service. In Italy, there were no differences in formal recommendations on testing strategies between children and adults and no screening in schools was recommended. In practice, in the context of school outbreak investigations, students may have a higher probability of being tested for SARS-CoV-2 than the general population, even in the absence of symptoms.

The vaccination date for each dose was retrieved by the vaccination registry of the local health authority of Reggio Emilia. The local vaccination registry included all the vaccination doses provided within the vaccination hub in the Reggio Emilia province since the beginning of the vaccination campaign. In Italy, the distribution of the COVID-19 vaccine began on 31 December 2020, and initially targeted healthcare professionals, police and public service forces, teachers and people aged 80 years or older. From 12 March 2021, the target of the national vaccination campaign was expanded to other categories, prioritising frail people with predefined comorbidities and age categories (i.e. 70–79 years, 60–69 years, and, successively, 18–60 years). Vaccination for adolescents aged 12–17 years started on 11 August 2021, while for children aged 5–11 years, the campaign started in the middle of December 2021. Of the vaccination doses included in the study, 90.0% were COVID-19 mRNA vaccines Comirnaty (BNT162b2 mRNA, BioNTech-Pfizer) and Spikevax (mRNA-1273, Moderna) while the reaming where viral vector vaccines Vaxzevria (ChAdOx1 nCoV-19, Oxford-AstraZeneca) and Janssen vaccine (Ad26.COV2-S, Janssen-Cilag International NV) mainly used for first and second doses. Comorbidities were retrieved by the hospital records, cancer and diabetes registries of the local health authority of Reggio Emilia [17]. Data from the hospital records and the mortality database of the local health authority of Reggio Emilia were also used to assess hospitalisation and death. Data sources were linked using the fiscal ID. A detailed description of data sources is presented in Supplementary Material - Data sources.

### Exposure and endpoints

In the main analysis, exposure was defined as having a documented prior SARS-CoV-2 infection.

A ‘first infection’ was defined as a person testing positive for SARS-CoV-2 for the first time with a valid test. Exposed at-risk person-time started 90 days after the date of the first positive valid test for each individual. A ‘reinfection’ was defined as a person testing positive more than 90 days after the date of the first infection.

This time span has been used in the definition of reinfection provided by the Italian Ministry of Health that was comparable with that reported by the European Centre for Disease Prevention and Control (ECDC) for other countries [18,19]. This definition aimed to distinguish real reinfections from tests that were positive because of non-viable SARS-CoV-2 genetic material that may last for months after recovery from a prior infection, even after a negative test [8,20]. 

The primary study endpoint was a SARS-CoV-2 infection documented in the local COVID-19 surveillance registry, including all tests performed in the Reggio Emilia province from 20 February 2020 up to 28 February 2022. All first infections and second infections (i.e. reinfections) were included. Third infections (i.e. second reinfections) were excluded from the study. The study flowchart is shown in Supplementary Figure S1. A secondary endpoint was severe disease, defined COVID-19 requiring hospitalisation within 28 days from diagnosis, or death within 90 days. Death within 90 days after a SARS-CoV-2 infection was also considered as a separate secondary endpoint. SARS-CoV-2-infected individuals who did not need emergency or hospital care and who did not die were classified in the category ‘No admission to emergency department or hospital’.

### Follow-up

The study follow-up started on 20 February 2020 and ended on 28 February 2022. At-risk person-time for a first infection started on 20 February 2020, while person-time at risk of re-infection started 90 days after the date of the first infection for each individual. At-risk person-time ended on the date of occurrence of the study’s endpoint (i.e. reinfection), death or end of follow-up, whichever came first.

Two secondary analyses excluding or including only the Omicron BA.1-driven wave were performed, ending the follow-up on 20 December 2021 for the former and starting the follow-up on 1 January 2022 for the latter. The study design was reported in Supplementary Figure S2 Study timeline. The period between 21–31 December 2021 (transition phase) was excluded from secondary analyses.

To assess disease severity, hospitalisation was attributed to a SARS-CoV-2 infection if the hospital admission occurred from 3 days before up to 28 days after the date of diagnosis (i.e. positive test). Death was attributed to a SARS-CoV-2 infection if occurring within 90 days from diagnosis and fulfilled the criteria for reporting COVID-19 as the main cause of death [21]. Thus, COVID-19-related hospitalisations were assessed up to 28 March 2022, while COVID-19-related deaths were assessed up to 28 May 2022.

### Covariates

The risk of infection was assessed taking simultaneously into account sex (collected as a binary variable), age, vaccination history, comorbidities reported as Charlson comorbidity index (CCI), prior SARS-CoV-2 infection, time from the first diagnosis, severity of the first disease and SARS-CoV-2 variant of the first infection [22]. Exposure to prior SARS-CoV-2 infection and vaccination status were the only time-dependent variables and were assessed on a daily basis; no lag time was considered for vaccination status. All other variables were assessed at the beginning of the study period (i.e. 20 February 2020).

### Statistical analysis

Descriptive analysis of the study cohort including sex, age, vaccination status, and CCI overall and by SARS-CoV-2 infection history were reported. For COVID-19 cases, the viral variant and disease severity attributed to each SARS-CoV-2 infection were also shown.

Vaccination coverage overtime was calculated as the percentage of people who received a first, second and third COVID-19 vaccination dose. In this study, the third dose was considered a booster. According to the vaccination schedule, the third dose was part of the primary vaccination cycle only for immunocompromised patients. However, we were unable to identify the immunocompromised patients in the analysis, and given the limited number, the complete schedule was defined to be two doses.

Cox proportional hazards models were used to estimate hazard ratios (HR) with 95% confidence intervals (95% CI) for SARS-CoV-2 infection by vaccination history and by prior SARS-CoV-2 infection, severity of the first diagnosis (hospitalised or not), time elapsed since first diagnosis (in months) and epidemic phase (wild-type, Alpha, Delta and Omicron BA.1). Models were adjusted for sex, age, CCI and vaccination history.

Cox proportional hazards models were performed including the pre-Omicron and Omicron BA.1 period separately. Cox proportional models by period including a test for interaction between prior infection and vaccination were also performed to assess the potential synergic effect of the combination of the two. Models were adjusted for sex, age and CCI. A sensitivity analysis including only adults (i.e. people aged ≥ 18 years) or only people aged ≤ 17 years were also performed.

Regarding the risk of severe disease and death, multivariable analysis was performed using a logistic regression model to measure the odd ratios (OR), with the relative 95% CI, for reinfections compared with first infections, adjusting for age, sex, vaccination history, CCI and stratifying by pre-Omicron and Omicron BA.1 pandemic period. Infections occurring before 31 August 2020, were excluded from the analysis on disease severity since most cases of COVID-19 during the first wave were diagnosed within hospital settings in people with moderate-to-severe symptoms, resulting in a strong underestimation of mild and asymptomatic cases. Logistic regression models were also performed to measure the OR of severe disease and death, with relative 95% CI, by immunisation status, adjusting for age, sex, vaccination history, CCI and stratifying by pre-Omicron and Omicron pandemic period. Logistic regression models including a test for interaction between vaccination and prior infection by study period were also performed. Absolute risk and risk difference of severe disease and death adjusting for age, sex, vaccination history, and CCI were also calculated together with the 95% CI obtained from the exact binomial distribution.

STATA v. 16.0 was used for all analyses (StataCorp LLC).

## Results

The cohort included 538,516 residents, 121,154 (22.5%) of whom were diagnosed with a first SARS-CoV-2 infection by the end of February 2022. The individuals who were reinfected (n = 3,739) had an average age of 33.8 years (standard deviation (SD): 19.9) with 46.3% male and 38.5% unvaccinated. The median time between the first and second infections was 362 days (interquartile range (IQR): 300–429). Only seven second reinfections were detected in our study, of which two occurred during the transition phase and five during the Omicron BA.1 period. Second reinfections were not included in this analysis. The overall cumulative incidence of reinfection was 3.1%. Of the total, 86.7% (3,243/3,739) of reinfections occurred in the period dominated by the Omicron BA.1 variant. Hospitalisation within 28 days and death within 90 days occurred in 1.3% and 0.1% of reinfections, respectively. Among first infections, hospitalisation occurred in 3.4% of cases, while death in 1.2% (Table 1). In the same period, the all-cause mortality among the included population was 2.1%.

**Table 1:** Cohort characteristics overall and by disease history, Reggio Emilia province, Italy, 20 February 2020–28 February 2022 (n = 538,516 individuals)

Variables	Population (n = 538,516)		SARS-CoV-2 testing					
			Individuals without a positive test (n = 417,362)		Individuals with a confirmed first infection (n = 121,154)		Individuals with a confirmed reinfection (n = 3,739)	
	n	%	n	%	n	%	n	%
**Age group (years)**								
0–4	21,251	3.9	15,036	3.6	6,215	5.1	124	3.3
5–11	37,701	7.0	24,634	5.9	13,067	10.8	414	11.1
12–19	43,283	8.0	30,039	7.2	13,244	10.9	541	14.5
20–49	203,312	37.8	150,603	36.1	52,709	43.5	1,905	50.9
50–64	116,209	21.6	94,654	22.7	21,555	17.8	510	13.6
65–79	78,477	14.6	69,067	16.5	9,410	7.8	117	3.1
≥ 80	38,283	7.1	33,329	8.0	4,954	4.1	128	3.4
Total (mean (SD))	44.3 (23.5)		46.3 (23.4)		37.3 (22.3)		33.8 (19.9)	
**Sex **								
Male	265,395	49.3	206,177	49.4	59,218	48.9	1,732	46.3
Female	273,121	50.7	211,185	50.6	61,936	51.1	2,007	53.7
**Vaccination status **								
Unvaccinated	102,631	19.1	73,525	17.6	29,106	24.0	1,441	38.5
Vaccinated with one dose	14,661	2.7	5,559	1.3	9,102	7.5	1,103	29.5
Vaccinated with two doses	109,001	20.2	53,729	12.9	55,272	45.6	891	23.8
Vaccinated with three doses	312,223	58.0	284,549	68.2	27,674	22.8	304	8.1
**Charlson Comorbidity Index **								
0	485,921	90.2	372,992	89.4	112,929	93.2	3,496	93.5
1	30,936	5.7	26,185	6.3	4,751	3.9	126	3.4
2	13,550	2.5	11,359	2.7	2,191	1.8	66	1.8
3	8109	1.5	6826	1.6	1,283	1.1	51	1.4
**Epidemic phase **								
Wild-type (20 Feb 2020–31 Dec 2020)	NA				22,596	18.7	28	0.7
Alpha (1 Jan–30 Jun 2021)					21,684	17.9	56	1.5
Delta (1 Jul–20 Dec 2021)					10,452	8.6	90	2.4
Transition (21–31 Dec 2021)					8,762	7.2	322	8.6
Omicron BA.1 (1 Jan 2022–28 Feb 2022)					57,660	47.6	3,243	86.7
**Disease severity**								
No admission to emergency department or hospital	NA				115,564	95.4	3,685	98.6
Hospitalisation					4,100	3.4	49	1.3
Death					1,490	1.2	5	0.1

SD: standard deviation; NA: not applicable; SARS-CoV-2: severe acute respiratory syndrome coronavirus 2.

Disease severity: hospitalisation was attributed to a SARS-CoV-2 infection if occurring from 3 days before up to 28 days after the date of diagnosis (i.e. positive test), while death was attributed if occurring within 90 days from diagnosis.

**Figure 1:**
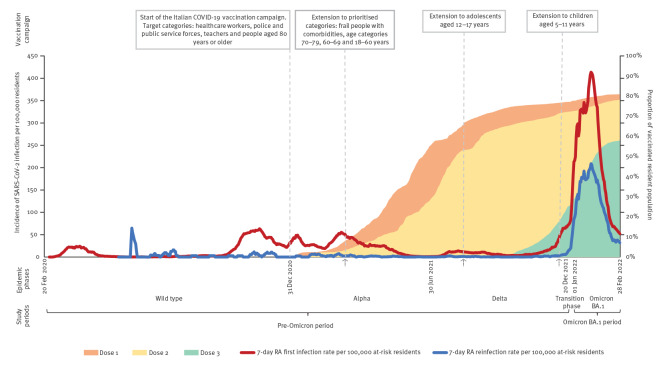
Incidence rate of first SARS-CoV-2 infections and reinfections and COVID-19 vaccination coverage in the study population, Reggio Emilia province, Italy, 20 February 2020–28 February 2022 (n = 538,516 individuals)

COVID-19: coronavirus disease; RA: rolling average; SARS-CoV-2: severe acute respiratory syndrome coronavirus 2.

The rolling average per 100,000 people of first infections and reinfections incidence in the Reggio Emilia population varied greatly across the study period, depicted by a four-wave epidemic curve with the highest increase during the Omicron BA.1 driven wave, despite the high vaccination coverage achieved in 2021 (Figure 1). 

The epidemic phases named using the predominant SARS-CoV-2 variant of each phase, the study periods and the roadmap of the Italian vaccination campaign are shown. Residents at-risk for first infection were those without a prior infection (i.e. naive). Residents at-risk for reinfection were those with a prior infection occurring more than 90 days before.

### Type of immunity and risk of infection

The adjusted risk of infection differed substantially between the pre-Omicron and Omicron BA.1 periods, in all strata by natural, vaccine-induced and hybrid immunity (Table 2).

**Table 2:** Risk of SARS-CoV-2 infection by immunisation status, adjusted for sex, age and Charlson Comorbidity Index in the pre-Omicron and Omicron BA.1 periods, Reggio Emilia province, Italy, 20 February 2020–28 February 2022 (n = 538,516 individuals)

Covariates	Period of SARS-CoV-2 infection							
	Pre-Omicron 20 Feb 2020–20 Dec 2021				Omicron BA.1 1 Jan–28 Feb 2022			
	Persons-days	Infections	HR	95% CI	Persons-days	Infections	HR	95% CI
**Immunisation status **								
No infection, no vaccine	263,722,053	48,453	1	NA	4,736,452	16,222	1	NA
No infection, 1 dose	15,548,031	1,568	0.68	0.65–0.72	576,317	2,502	1.15	1.11–1.20
No infection, 2 doses	57,753,826	4,449	0.33	0.32–0.34	5,995,660	24,075	1.13	1.11–1.16
No infection, 3 doses	3,275,147	262	0.11	0.10–0.13	14,399,876	14,861	0.47	0.45–0.48
Infection, no vaccine	5,745,099	135	0.10	0.09–0.12	392,080	1,311	0.91	0.86–0.96
Infection, 1 dose	3,966,986	23	0.02	0.02–0.04	574,413	923	0.44	0.41–0.47
Infection, 2 doses	2,039,503	15	0.02	0.01–0.04	1,177,511	741	0.24	0.23–0.26
Infection, 3 doses	73,504	1	0.02	0.00–0.12	514,457	268	0.22	0.20–0.25
**Sex**								
Male	173,555,410	27,055	1	NA	13,998,026	29,319	1	NA
Female	178,568,739	27,851	1.02	1.01–1.04	14,368,740	31,584	1.12	1.10–1.14
**Age group (years) **								
0–4	14,074,477	1,803	1	NA	1,095,032	4,268	1	NA
5–11	24,875,445	4,750	1.56	1.48–1.64	1,861,123	7,883	1.10	1.06–1.14
12–19	28,484,072	5,706	1.91	1.81–2.01	2,205,844	6,941	1.01	0.97–1.05
20–49	134,112,025	22,364	1.60	1.53–1.68	10,669,146	27,704	0.88	0.85–0.91
50–64	76,559,955	11,144	1.42	1.35–1.49	6,393,687	9,371	0.58	0.56–0.61
65–79	51,165,245	5,607	1.07	1.01–1.13	4,349,740	3,320	0.34	0.32–0.36
≥ 80	22,852,930	3,532	1.63	1.54–1.73	1,792,194	1,416	0.39	0.36–0.41
**Charlson Comorbidity Index **								
0	319,459,054	49,755	1	NA	25,725,219	57,959	1	NA
1	19,630,453	2,947	1.18	1.13–1.22	1,620,196	1,706	0.96	0.91–1.01
2	8,401,431	1,311	1.23	1.16–1.30	673,997	830	1.16	1.08–1.24
3	4,633,211	893	1.56	1.46–1.68	347,354	408	1.23	1.11–1.36

CI: confidence interval; HR: hazard ratio; NA: not applicable; SARS-CoV-2: severe acute respiratory syndrome coronavirus 2.

In the pre-Omicron period, booster vaccination in I individuals reduced the risk of infection by 89% (HR: 0.11; 95% CI: 0.10–0.13) while natural immunity without vaccination reduced the risk of reinfection by 90% (HR: 0.10; 95% CI: 0.09–0.12). The hybrid immunity coming from booster vaccination and prior infection further reduced the risk (HR: 0.02; 95% CI: 0.00–0.12). The association of prior infection and vaccination resulted in protection stronger than the multiplicative combination of hazard ratios, with a statistically significant test for interaction (interaction term, HR: 0.67, 95% CI: 0.47–0.96; p = 0.030).

During the Omicron BA.1-driven wave, the overall protection was lower. A booster dose of vaccine in I people reduced the risk by 53% (HR: 0.47; 95% CI: 0.45–0.48), while naturally acquired immunity protection was trivial in reducing risk of infection (HR: 0.91; 95% CI: 0.86–0.96). Nevertheless, hybrid immunity showed a 56% risk reduction (HR: 0.44; 95% CI: 0.41–0.47) with one dose of vaccine, rising to 76% and 78% protection with 2 and 3 doses, respectively. This suggests a synergic effect of prior infection and vaccination (interaction term, HR: 0.46, 95% CI: 0.43–0.49; p < 0.001).

In the sensitivity analyses stratified by age (i.e. < 18 and ≥ 18 years), the estimates of protection were consistent between the two age strata in the pre-Omicron period, while it differed substantially during the Omicron BA.1 period. Compared with unvaccinated uninfected individuals, a prior infection provided 43% more protection against reinfection in people aged < 18 (HR: 0.57, 95% CI: 0.52–0.61) while a 57% increased risk was found in previously infected adults (HR: 1.57; 95% CI: 1.45–1.69). The results of sensitivity analysis stratified by age (i.e. < 18 and ≥ 18 years) are reported in Supplementary Tables S1 and S2.

### Determinants of naturally acquired protection from the risk of reinfection

Considering age, sex, vaccination status and comorbidities, the overall protection from reinfection of naturally acquired immunity was 92% (HR: 0.08; 95% CI: 0.07–0.10) in the pre-Omicron phase and 58% (HR: 0.42; 95% CI: 0.41–0.44) during the Omicron BA.1-driven wave (Table 3).

**Table 3:** Risk of SARS-CoV-2 infection by disease history, severity of the first infection, and epidemic phase of the first infection in the pre-Omicron and Omicron BA.1 periods, Reggio Emilia province, Italy, 20 February 2020–28 February 2022 (n = 538,516 individuals)

Cox proportional hazards models	Period of SARS-CoV-2 infection			
	Pre-Omicron 20 Feb 2020–20 Dec 2021		Omicron BA.1 1 Jan–28 Feb 2022	
	HR	95% CI	HR	95% CI
**Model 1: Disease history**				
No prior infection	1	NA	1	NA
Prior infection	0.08	0.07–0.10	0.42	0.41–0.44
**Model 2: Severity of the first infection**				
No prior infection	1	NA	1	NA
Prior infection, no hospitalisation	0.08	0.07–0.10	0.43	0.41–0.45
Prior infection, hospitalisation	0.11	0.07–0.18	0.29	0.24–0.36
**Model 3: Epidemic phase**				
No prior infection	1	NA	1	NA
Prior infection, wild-type	0.09	0.08–0.11	0.49	0.46–0.51
Prior infection, Alpha	0.07	0.05–0.09	0.38	0.36–0.40
Prior infection, Delta	0.03	0.01–0.12	0.37	0.33–0.42

CI: confidence interval; HZ: hazard ratio; NA: not applicable; SARS-CoV-2: severe acute respiratory syndrome coronavirus 2.

All models are adjusted by age, sex, number of COVID-19 vaccination doses and Charlson Comorbidity Index.

The severity of the first infection was associated with protection from reinfections only during the Omicron BA.1 phase of the epidemic (71% vs 57% risk reduction in hospitalised and non-hospitalised COVID-19 patients, respectively, compared with uninfected individuals), while in the pre-Omicron period no increase of protection was observed (Table 3). 

**Figure 2:**
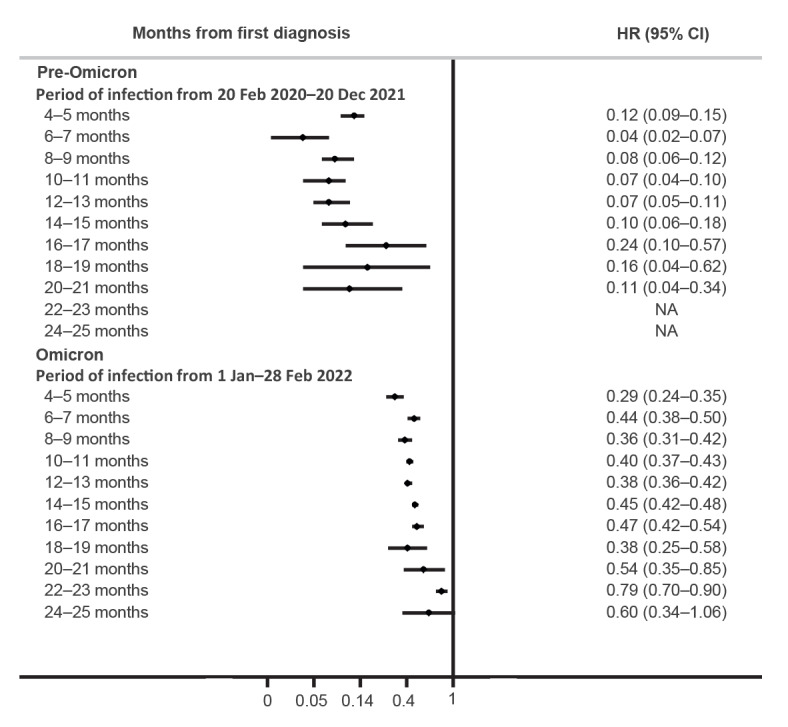
Risk of SARS-CoV-2 infection by time from the first diagnosis compared with no infection in the pre-Omicron and Omicron BA.1 period, Reggio Emilia province, Italy, 20 February 2020–28 February 2022 (n = 538,516 individuals)

CI: confidence interval; HR: hazard ratio; NA: not applicable; SARS-CoV-2: severe acute respiratory syndrome coronavirus 2.

Considering the SARS-CoV-2 variant of first infection, compared with the wild-type, infections from Alpha and Delta variants showed slightly higher protection against Omicron BA.1 reinfections (62% and 63% vs 51%, respectively) (Table 3). Considering the time from the first infection, evidence of natural immunity protection against reinfection persists for up to 23 months, although showing a declining trend, especially during the Omicron BA.1 phase (Figure 2).

Cox regression model is adjusted by age, sex, number of COVID-19 vaccination doses and Charlson Comorbidity Index.

### Severity of reinfection

Compared with first infections, reinfections showed a lower risk of severe disease (i.e. including hospitalisation and/or death) in the Omicron BA.1 period (HR: 0.62; 95% CI: 0.43–0.90), considering sex, age, vaccination history and CCI. In the pre-Omicron, the estimate was less precise and suggests limited protection of prior infection, if any, against severe disease (HR: 0.79; 95% CI: 0.42–1.47) (Table 4). The absolute numbers of events included in the models were reported in Supplementary Table S3. The risks of severe infection and death by immunisation status were also assessed and reported in Supplementary Table S4. The models did not provide any estimate for protection against death in almost all the hybrid immunity combinations, and the precision of other estimates substantially decreased. While models provided estimates of protection against severe disease, even if with large confidence intervals, the results suggest a protective effect of prior infection in unvaccinated persons in the Omicron BA.1 period only. The protection from hybrid immunity against severe diseases was consistent with a multiplicative model summing the effect of vaccine and prior infection in both pre-Omicron and Omicron BA.1 periods, with no interaction (interaction term, OR: 0.74, 95% CI: 0.18–3.00; p = 0.674, and OR: 1.64, 95% CI: 0.72–3.72; p = 0.237, respectively).

**Table 4:** Odds ratios of severe disease and death from COVID‐19 for reinfections (n = 3,739) vs first infections (n = 121,154), Reggio Emilia province, Italy, 20 February 2020–28 February 2022

Covariates	Period of infection							
	Pre-Omicron 1 Sep 2020–20 Dec 2021^a^				Omicron BA.1 1 Jan–28 Feb 2022			
	Severe disease^b^		Death		Severe disease^b^		Death	
	OR	95% CI	OR	95% CI	OR	95% CI	OR	95% CI
**Exposure **								
First infection	1	NA	1	NA	1	NA	1	NA
Reinfection	0.79	0.42–1.47	0.74	0.20–2.74	0.62	0.43–0.90	0.06	0.01–0.48
**Sex **								
Male	1	NA	1	NA	1	NA	1	NA
Female	0.64	0.60–0.70	0.5	0.43–0.59	0.79	0.67–0.93	0.49	0.33–0.73
**Age**								
All residents^c^	1.07	1.07–1.07	1.14	1.13–1.14	1.08	1.07–1.08	1.19	1.16–1.21
**Vaccination status **								
Unvaccinated	1	NA	1	NA	1	NA	1	NA
Vaccinated with one dose	0.55	0.45–0.68	0.46	0.32–0.67	1.02	0.68–1.53	0.98	0.27–3.51
Vaccinated with two doses	0.32	0.28–0.37	0.38	0.28–0.51	0.4	0.32–0.49	0.48	0.26–0.88
Vaccinated with three doses	0.23	0.17–0.30	0.2	0.11–0.36	0.29	0.24–0.36	0.2	0.12–0.33
**Charlson Comorbidity Index **								
0	1	NA	1	NA	1	NA	1	NA
1	1.93	1.72–2.16	2.33	1.92–2.82	2.58	2.05–3.26	2.36	1.46–3.81
2	1.87	1.59–2.21	1.85	1.40–2.45	3.66	2.76–4.84	3.32	1.89–5.83
3	3.23	2.69–3.87	4.94	3.85–6.33	5.11	3.72–7.00	2.59	1.30–5.15

CI: confidence interval; COVID-19: coronavirus disease; OR: odds ratio; SARS-CoV-2: severe acute respiratory syndrome coronavirus 2.

^a^ The pre-Omicron period of infection in the present analysis started on 1 September 2020 because infections occurring before 31 August 2020 were excluded. This was done since most diagnosed cases of COVID-19 during the first wave were diagnosed within hospital settings in people with moderate to severe symptoms, resulting in a strong underestimation of mild and asymptomatic cases.

^b^ Including hospitalisation and/or death for COVID‐19.

^c^ OR for 1-year increase.

Odds ratios are adjusted for age, sex, vaccination history, Charlson Comorbidity Index and SARS-CoV-2 variant.

Results on relative risks of severe disease between first infections and reinfections should be interpreted considering absolute risks, which showed important differences as reported in Supplementary Table S5. During the pre-Omicron period, the overall risk of severe disease was 59.3 per 1,000 positive individuals (95% CI: 57.6 to 61.0) in first infections and 49.5 (95% CI: 25.6 to 73.5) in reinfections, corresponding to a risk difference of −9.8 severe cases per 1,000 positive individuals (95% CI: −16.8 to −2.7). During the Omicron BA.1 period, the overall risk of severe disease was 11.0 per 1,000 positive subjects (95% CI: 10.2 to 11.8) in first infections and 7.1 (95% CI: 4.7 to 9.5) in reinfections, corresponding to a risk difference of −3.9 severe diseases per 1,000 positive subjects (95% CI: –6.5 to −1.2).

Since infections occurring before 31 August 2020 were excluded from the analysis on disease severity, only four deaths related to reinfections were included, reducing the precision of protection estimates. The absolute numbers of severe diseases and deaths were reported in Supplementary Table S3.

A sensitivity analysis on infections occurring in a period when all random genotyping confirmed the Omicron BA.1 variant in our Province (i.e. after 15 January 2022) showed consistent results. The results of the sensitivity analysis were reported in Supplementary Table S6.

## Discussion

In the Reggio Emilia province, the cumulative incidence of detected SARS-CoV-2 infections in the study period, i.e. up to 28 February 2022, reached 23.2% while the cumulative overall incidence of reinfection was 3.08% of those at risk for reinfection. A prior infection gave 90% protection from reinfections until the spread of Omicron BA.1, when the protection decreased to ca 50%. Protection lasted for at least 23 months from first infection, even with an Omicron BA.1 infection, and slowly waned over time. Severe disease, i.e. requiring hospitalisation, increased protection for reinfection when the dominant virus variant was Omicron BA.1, but this effect was not significant when other variants were dominant. The protection against reinfection of natural immunity was similar between adults and those younger than 18 years in the pre-Omicron period, while differences were found during the Omicron BA.1 period. Nevertheless, all the analyses showed that vaccination was effective in reducing reinfection risk with a positive interaction between natural and vaccine-induced protection. Finally, reinfections were less severe than first infections with a 38% decrease in the probability of hospitalisation and death in the Omicron BA.1 period. Little, if any, reduction in disease severity was found for reinfections during the pre-Omicron period compared with first infections. Vaccination reduced disease severity in individuals with and without prior infection, yet no interaction was found between the two types of immunity against severe disease.

Three reviews, which include more than 35 studies published before October 2021, reported estimates of protection between 80% and 90% against reinfection by SARS-CoV-2 wild-type, Alpha, Beta, and Delta variants’ first infections [7,8,23]. These results are consistent with our findings in the pre-Omicron period. A narrative review published in February 2022, highlighted quite consistent results on limited, if any, waning of protection for at least 13 months from diagnosis, as confirmed by our results that showed a sustained effect up to 23 months [8].

Since May 2022 when this analysis was performed, a growing body of evidence has been published on Omicron variants. A recent meta-analysis including studies published up to June 2022 reported pooled estimates of protection against Omicron reinfection from natural immunity of 65.2% at 3 months, waning to 24.7% at 12 months [14]. No differences by age group or variant of first infection (Alpha, Delta or mixed variants) were found. In our study, the overall protection against Omicron BA.1 reinfection provided by prior infection was 9% and differed by age group. The pooled estimates produced by the review were consistent with our results on people under 18 years of age (43% protection), which was the sub-population most represented among unvaccinated individuals on 1 January 2022 in Italy. Among adults, we found a 52% increased risk of reinfection among those unvaccinated with prior infection compared with unvaccinated naive. We interpreted this counterintuitive result, which we did not find in the pre-Omicron period, in the light of the Italian SARS-CoV-2 control policy acting on this specific population in 2021. In 2021, the Italian Ministry of Health released the green pass certificate only to people who completed primary vaccination schedule in the previous 6 months, with a booster dose or who recovered from SARS-CoV-2 infection in the last 12 months, or with a negative COVID-19 test in the last 48–72 hours [24,25] From October 2021, the certificate became mandatory to attend any workplace and from December 2021, only certificates generated by vaccination or recovery from a previous infection were considered valid. Thus, a higher propensity to test and notification of case confirmation linked to the need for the green pass certificate may have led to a self-selection of unvaccinated adults with a prior infection among all unvaccinated adults at the beginning of 2022. This phenomenon may have also affected the estimates of protection from the first and second vaccination doses in the Omicron BA.1 period, although to a lesser extent, especially in people without prior infection, while no effect is expected in people with a booster dose or hybrid immunity with a complete vaccination cycle.

We explored the effect of severity of the first infection on risk of reinfections, finding a protective effect of severe disease only during the Omicron BA.1 period [7,8]. This finding should be interpreted considering the possible floor effect when looking for risk reduction given by prior infection against pre-Omicron variants in a highly vaccinated population. Regarding the severity of reinfections, a growing body of evidence on pre-Omicron variants (i.e. Alpha, Beta, Delta) shows a milder clinical course compared with primary infections with up to 90% reduction of hospitalisation risk [8]. Our findings showed only a slight, if any, reduction in disease severity of reinfections compared with first infections in the pre-Omicron period, while we found an overall 40% reduction in risk of severe disease in the Omicron BA.1 period. Stratifying by immunisation status, we found no protection against severe disease from prior infection among unvaccinated individuals in the pre-Omicron period and 44% in the Omicron BA.1 period. Vaccination resulted in additional protection against severe disease in both naive and previously infected individuals, even in the absence of a synergic effect. These findings differed from those reported in a recent study from Sweden showing a beneficial effect of natural protection against severe disease among unvaccinated individuals only, although in both studies the stratified estimates were highly imprecise [26]. On the contrary, our findings were consistent with the recent meta-analysis which reported more sustained protection against severe disease from hybrid immunity compared with immunity from prior infection only [14]. Moreover, in our study, the absolute risks of hospitalisation and death were much lower in the Omicron BA.1 period than in the pre-Omicron period for both first infections and reinfections.

The interplay of natural and vaccine-induced immunities and the role of cell-mediated immunity are debated in the literature [8,10]. In our study, the combination of vaccination and prior infection provided a gain in protection against reinfection in all the analyses. In the pre-Omicron period, the high protection conferred by natural immunity alone (90%) increased with the combination of one dose (98%), with no further gain with two or three doses, probably because of the floor effect. In the Omicron BA.1 period, our data showed that the combination of infection and two vaccine doses gives greater protection against reinfection than three doses of complete vaccination with booster (76% vs 53%, respectively). Similar findings came from health surveillance data in England showing greater protection from complete vaccination with infection (72%) than complete vaccination without having had the disease (62%) [12]. Our findings showed a lower waning than those reported by the systematic review of studies on Omicron reinfections [14].

Findings from our study and the recent meta-analysis provided epidemiological data consistent with findings from laboratory studies which claimed long-term and protective immunity by SARS-CoV-2 infection in addition to vaccination likely because of a higher capacity to trigger mucosal immunity, over the vaccination alone [27-29]. To shed light on the so-called ’hybrid immunity’, immunological studies were performed on the development of adaptive immune response in breakthrough SARS-CoV-2 infected individuals. The immune memory landscape of vaccinated, previously infected individuals is completely different from that of SARS-CoV-2-naive individuals, suggesting that the SARS-CoV-2 infection establishes multiple features of immune memory not only characterised by a strong type-1 antiviral immunity, but also by higher concentration of receptor-binding domain (RBD)-specific memory B-cells, plasmatic level of IgG- and IgA-neutralising antibodies and more diversified CD8+ T cell memory repertoire. [30,31]. This robust and specific antiviral immunity could explain the reason why they are more protected from infection, as suggested in our results. This model is also consistent with our finding that the severity of prior infection showed to increase the protection against reinfections, particularly for Omicron lineages. A promising indication of continued vaccine efficacy is the fact that repeated recalls induce a shift in immune memory phenotypes towards a terminally differentiated effector memory phenotype without leading to exhaustion [31]. The mechanisms underlying the high protection (more than multiplicative in Omicron BA.1) from the combination of vaccine and infection, and the effect of severity of prior infection on protection against reinfection should be investigated in future studies. Evidence stratified by age groups and variant of first infection is also needed.

Our study had some limitations. Firstly, the main intrinsic limitation of this study is that undetected SARS-CoV-2 infections may lead to a misclassification of the disease history of recruited people. Furthermore, the number of undetected SARS-CoV-2 infections have probably varied widely in different periods of the pandemic. Test availability, testing strategies and test-seeking behaviours differed substantially across the study period, introducing a selection bias, especially for patients diagnosed during the first wave of the pandemic. Test-seeking behaviours may also differ between population subgroups and by immunisation status as discussed above. Secondly, reinfections were classified based on laboratory findings without clinical assessment, leading to potential misclassification of cases with long-lasting SARS-CoV-2 RNA for over 90 days after a primary infection. Natural and vaccine-induced immunities were assessed based on disease history and COVID-19 vaccination uptake, respectively, without a direct measure of antibody response. We could not determine how the time elapsed since the last vaccine dose impacted the probability of reinfection. Indeed, as the number of values for the exposure variable be too high with limited person-time, attempting to do this would reduce the precision of the estimates. Thirdly, SARS-CoV-2 variants were classified according to the prevalence in each study period rather than on genomic sequencing of all the included samples. Nevertheless, data from typing show that the periods of codominance of different variants lasted a small amount of time. Fourthly, the period of Omicron dominance in our study may have some misclassification of first infections since a small proportion of Delta was still present, but is negligible for reinfections that were virtually all caused by Omicron BA.1 [5]. Because the follow-up period ended on 28 February 2022, our study did not provide evidence on Omicron lineages other than BA.1 nor protection against reinfections provided by Omicron first infections. Finally, the assessment of disease severity was based on all-cause hospitalisation data, leading to an overestimation of severity in SARS-CoV-2-positive patients hospitalised for other reasons. The overestimation may be higher for Omicron BA.1 infections, compared with previous variants, because of its higher incidence among both general and hospitalised populations.

## Conclusions

Strategies for reducing the risk of infection over a long period should consider the interaction between natural and vaccine-induced immunity in the light of the surge of new variants. Our results are consistent with the growing body of evidence suggesting the importance of vaccinating people with prior infection to reach herd immunity, effectively limiting the public health burden of new epidemic peaks. 

## Supplementary Data

624000 Supplement
