# Supplementary material for: Risk of SARS-CoV-2 reinfection by vaccination status, predominant variant and time from prior infection: a cohort study, Reggio Emilia province, Italy, February 2020 to February 2022
Source: Euro Surveill. 2023 Mar 30;28(13):2200494. doi: 10.2807/1560-7917.ES.2023.28.13.2200494 (PMC10064646; doi:10.2807/1560-7917.ES.2023.28.13.2200494)
Supplement: Supplement [file 22-00494_VENTURELLI_SUPPLEMENT.pdf]

This supplementary material is hosted by *Eurosurveillance* as supporting information alongside the article "Risk of SARS-CoV-2 reinfection by vaccination status, predominant variant, and time from prior infection: a cohort study, Reggio Emilia province, Italy, February 2020 to February 2022", on behalf of the authors, who remain responsible for the accuracy and appropriateness of the content. The same standards for ethics, copyright, attributions and permissions as for the article apply. Supplements are not edited by Eurosurveillance and the journal is not responsible for the maintenance of any links or email addresses provided therein.

Supplementary Methods, Tables, and Figures – Risk of SARS-CoV-2 reinfection by vaccination status, predominant variant, and time from prior infection: a cohort study, Reggio Emilia province, Italy, February 2020 to February 2022

Supplementary material - Data sources

The residency status, age and sex where retrieved by the Population Registry of the Local Health Authority of Reggio Emilia.

The date of SARS-CoV-2 diagnosis and of COVID-19 related death were retrieved from the COVID-19 Surveillance Registry, coordinated by the Italian National Institute of Health, and implemented in each Local Health Authority. The whole list of cases tested for SARS-CoV-2 (positive and negative) from February 20, 2020, up to February 28, 2022, was linked with the resident population registry of the Local Health Authority COVID-19-related death were assessed up to May 28, 2022, to allow a 90-day follow-up for all the COVID-19 cases.

Hospitalization was retrieved by the Local Health Authority hospital records database.

Date of death related to causes other than COVID-19 was assessed through the Mortality Registry of the Local Health Authority of Reggio Emilia, including data up to December 31, 2021. Data on inpatients' all-cause related deaths were also collected from hospital discharge databases up to February 28, 2022.

COVID-19 vaccination status including the administration date of each dose and the type of vaccine was retrieved from the Vaccination Registry of the Local Health Authority of Reggio Emilia, as of February 28, 2022. The Vaccination Registry collects all vaccinations administered by the health services of the Local Health Authority. Vaccinations of residents administered outside the Local Health Authority were not registered, leading to a slightly underestimation of the actual vaccination coverage.

The SARS-CoV-2 variant of infection was attributed using temporal criteria of occurrence, splitting the study period according to the genotyping results of flash surveys conducted in the Emilia-Romagna region within the Italian National Institute of Health surveillance programme (https://www.epicentro.iss.it/coronavirus/sars-cov-2-monitoraggio-varianti-indagini-rapide). A dominant variant was attributed to each period as follows: Wild type from February 20, 2020, to December 31, 2020, Alpha from January 01, 2021, to June 30, 2021, Delta from July 1, 2021, to December 20, 2021, and Omicron BA.1 from January 01, 2022, to February 28, 2022. The period between December 21 and 31, 2021, was defined as a Transition phase since it is characterised by a concurrent circulation of Delta and Omicron BA.1 variants.

The impact of pre-existing comorbidities was assessed using the Charlson comorbidity index (CCI) CCI was calculated including all information on comorbidities retrieved from the hospital discharge databases, Diabetes registry, and Cancer Registry.[1, 2] Diabetes status was retrieved from the local Diabetes Registry, including all diabetes diagnoses among the residents of the Reggio Emilia province prevalent on December 31, 2019. The methods applied to set up the diabetes registry have been described previously.[1] The cancer diagnoses were assessed through linkage with two sources: hospital discharge databases and the local Cancer Registry. The local Cancer Registry included all cancer diagnoses occurring in people residing in the Reggio Emilia Province, from 2015 to 2019.[1] Information on other comorbidities was collected from hospital discharge databases, for the period 2015-2019.

Supplementary material - Data sources References:

1. Mangone L, Gioia F, Mancuso P, et al. Cumulative COVID-19 incidence, mortality and prognosis in cancer survivors: A population-based study in Reggio Emilia, Northern Italy [published online ahead of print, 2021 Apr 16]. Int J Cancer 2021;149(4):820-826. doi:10.1002/ijc.33601
2. Charlson ME, Pompei P, Ales KL, MacKenzie CR. A new method of classifying prognostic comorbidity in longitudinal studies: development and validation. J Chronic Dis 1987;40(5):373-383. doi:10.1016/0021-9681(87)90171-8

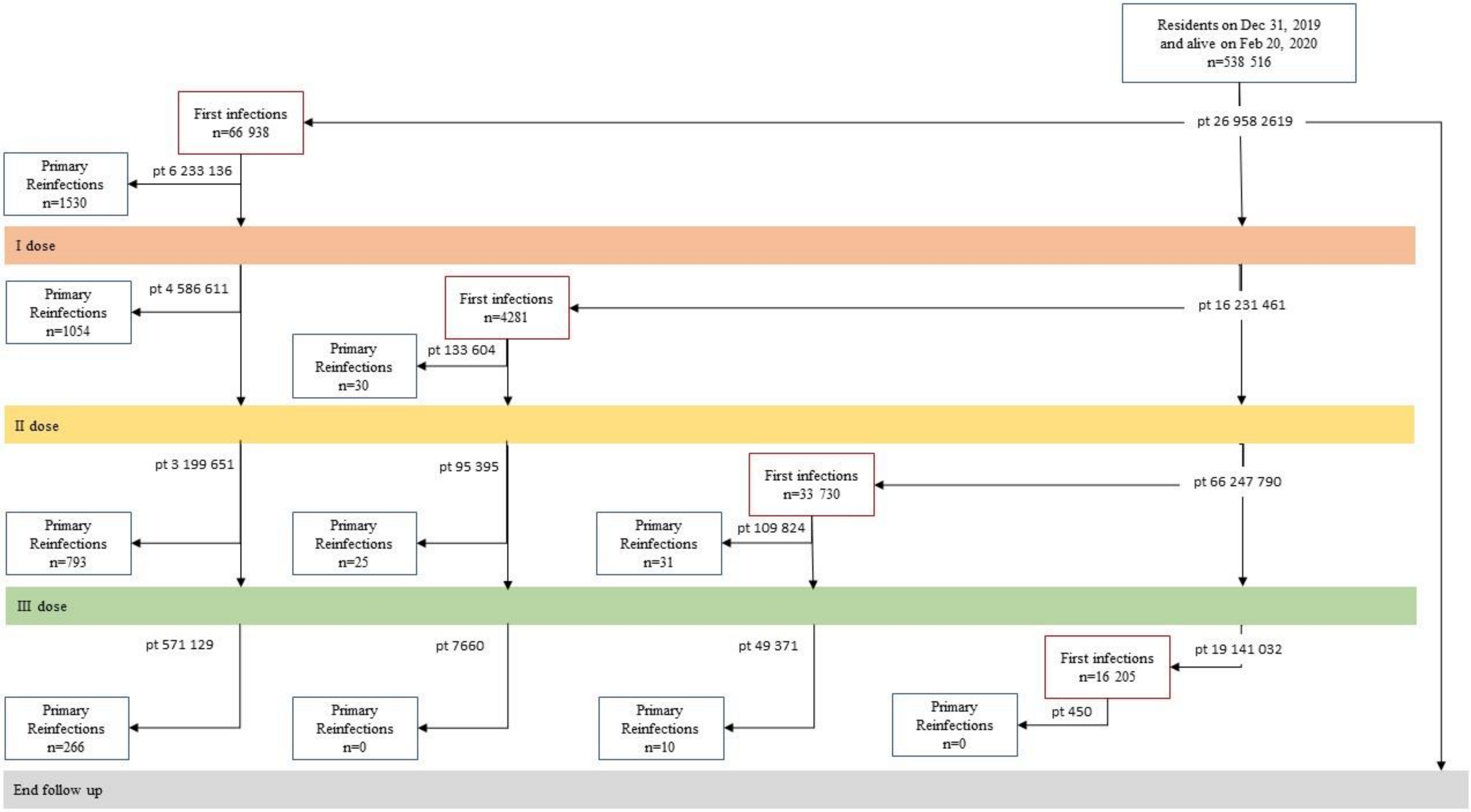

Supplementary Figure S1. Study Flow chart; pt= person time reported as person-day.

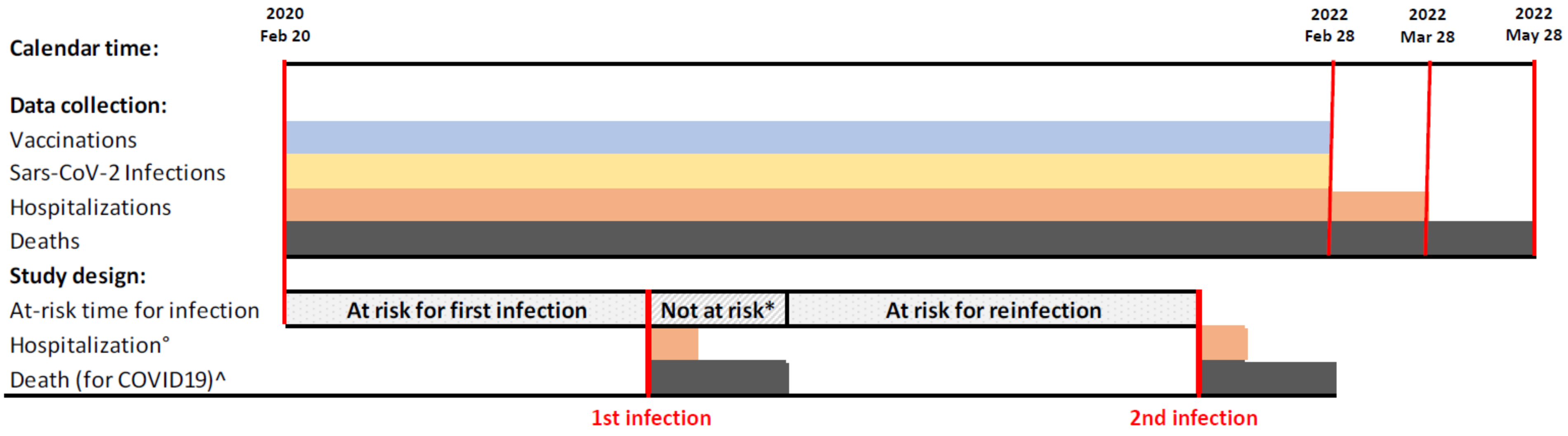

* Not at risk time last 90 days after the 1st positive test
° Infection related hospitalization was assessed from 3 days before to 28 days after the diagnosis
^ Infection related death was assessed up to 90 days after the first positive test
Sex and age were collected on Feb 20, 2020.
Charlson Comorbidity Index was assessed on data from 2015-2019.

Supplementary Figure S2. Study timeline.

	Period of infection<br>Feb 20, 2020 to Dec 20, 2021					Period of infection<br>Jan 01, 2022 to Feb 28, 2022				
	**Persons-days**	**Infections**	**HR**	**95%CI**		**Persons-days**	**Infections**	**HR**	**95%CI**	
**Immunization status**										
No infection, no vaccine	209,437,458	38,218	1			2,739,193	6789	1		
No infection, 1 dose	14,639,610	1380	0.70	0.66	0.75	266,869	840	1.21	1.13	1.30
No infection, 2 doses	54,321,060	4113	0.36	0.35	0.38	4,940,793	19,273	1.29	1.26	1.33
No infection, 3 doses	3,265,923	261	0.12	0.11	0.14	13,641,390	14,007	0.55	0.53	0.56
Infection, no vaccine	4,310,184	95	0.10	0.08	0.13	175,752	727	1.57	1.45	1.69
Infection, 1 dose	3,632,845	16	0.02	0.01	0.03	444,704	768	0.57	0.53	0.61
Infection, 2 doses	1,914,755	15	0.03	0.02	0.05	1,051,108	658	0.29	0.27	0.31
Infection, 3 doses	73,086	1	0.02	0.00	0.13	481,647	261	0.27	0.24	0.31
**Sex**										
Male	142,330,229	21,364	1			11,612,965	20,239	1		
Female	149,264,692	22,735	1.04	1.02	1.06	12,128,491	23,084	1.18	1.15	1.20
**Age group**										
18-19	6,904,766	1452	1			536,689	1512	1		
20-49	134,112,025	22,364	0.78	0.74	0.83	10,669,146	27,704	0.96	0.91	1.01
50-64	76,559,955	11,144	0.69	0.65	0.73	6,393,687	9371	0.63	0.60	0.67
65-79	51,165,245	5607	0.52	0.49	0.55	4,349,740	3320	0.37	0.34	0.39
80+	22,852,930	3532	0.78	0.73	0.83	1,792,194	1416	0.41	0.38	0.45
**Charlson Comorbidity Index**										
0	259,488,663	39,047	1			21,143,184	40,521	1		
1	19,218,682	2875	1.18	1.14	1.23	1,588,070	1598	0.97	0.92	1.02
2	8,279,281	1289	1.23	1.16	1.30	664,596	800	1.16	1.09	1.25
3	4,608,295	888	1.56	1.46	1.68	345,606	404	1.24	1.13	1.37

Supplementary Table S1. Sensitivity analysis of the risk of SARS-CoV-2 infection by immunization status, adjusted for sex, age and Charlson Comorbidity Index, in the pre-Omicron and Omicron BA.1 periods, including adults only (aged 18 or older). The pre-Omicron period was the period from February 20, 2020, to December 20, 2021, while the Omicron BA.1 period was the period from January 01, 2022, to February 28, 2022.

	Period of infection Feb 20, 2020 to Dec 20, 2021					Period of infection Jan 01, 2022 to Feb 28, 2022				
	Persons-days	Infections	HR	95%CI		Persons-days	Infections	HR	95%CI	
**Immunization status**										
No infection, no vaccine	54,284,595	10,235	1			1,997,259	9433	1		
No infection, 1 dose	908,421	188	0.90	0.78	1.05	309,448	1662	1.04	0.99	1.10
No infection, 2 doses	3,432,766	336	0.24	0.21	0.27	1,054,867	4802	0.83	0.79	0.86
No infection, 3 doses	9224	1	0.09	0.01	0.64	758,486	854	0.25	0.23	0.27
Infection, no vaccine	1,434,915	40	0.11	0.08	0.14	216,328	584	0.57	0.52	0.61
Infection, 1 dose	334,141	7	0.05	0.03	0.11	129,709	155	0.22	0.19	0.26
Infection, 2 doses	124,748	0	-	-	-	126,403	83	0.14	0.11	0.17
Infection, 3 doses	418	0	-	-	-	32,810	7	0.05	0.02	0.10
**Sex**										
Male	31,225,181	5691	1			2,385,061	9080	1		
Female	29,304,047	5116	0.96	0.92	1.00	2,240,249	8500	1.00	0.97	1.03
**Age group**										
0-4	14,074,477	1803	1			1,095,032	4268	1		
5-11	24,875,445	4750	1.57	1.49	1.66	1,861,123	7883	1.20	1.16	1.25
12-17	21,579,306	4254	1.93	1.82	2.04	1,669,155	5429	1.39	1.33	1.46
**Charlson Comorbidity Index**										
0	59,970,391	10,708	1			4,582,035	17,438	1		
1	411,771	72	0.99	0.79	1.25	32,126	108	0.92	0.76	1.11
2	122,150	22	0.99	0.65	1.50	9401	30	0.89	0.63	1.27
3	24,916	5	1.12	0.47	2.65	1748	4	0.61	0.22	1.68

Supplementary Table S2. Sensitivity analysis of the risk of SARS-CoV-2 infection by immunization status, adjusted for sex, age and Charlson Comorbidity Index, in the pre-Omicron and Omicron BA.1 periods, including people aged ≤17 years only. The pre-Omicron period was the period from February 20, 2020, to December 20, 2021, while the Omicron BA.1 period was the period from January 01, 2022, to February 28, 2022.

	Period of infection Feb 20, 2020 to Aug 31, 2020				Period of infection Sep 01, 2020 to Dec 20, 2021				Period of infection Jan 01, 2022 to Feb 28, 2022			
	**Severe disease**		**Death**		**Severe disease**		**Death**		**Severe disease**		**Death**	
	**No**	**Yes**	**No**	**Yes**	**No**	**Yes**	**No**	**Yes**	**No**	**Yes**	**No**	**Yes**
**Overall**	3595	1500	4512	583	55,404	3491	58,098	797	60,250	653	60,788	115
**Exposure**												
First infection	3589	1493	4500	582	54,934	3478	57,618	794	57,041	619	57,546	114
Re-infection	6	7	12	1	470	13	480	3	3209	34	3242	1
**Sex**												
Male	1471	817	1991	297	27,420	1923	28,907	436	28,986	333	29,258	61
Female	2124	683	2521	286	27,984	1568	29,191	361	31,264	320	31,530	54
**Age (mean) (SD)**	52.7 (21.6)	72.8 (15.8)	55.5 (21.3)	82.8 (10.0)	37.2 (21.1)	67.3 (17.3)	38.4 (21.6)	81.1 (10.6)	33.3 (20.8)	66.3 (22.6)	33.6 (21.0)	83.5 (8.4)
**Vaccination status**												
Unvaccinated	366	689	472	583	10,862	1111	11,278	695	17,335	184	17,490	29
Vaccinated with one dose	63	11	74	0	6415	292	6670	37	3392	32	3421	3
Vaccinated with two doses	479	89	568	0	29,175	1659	30,780	54	24,606	155	24,740	21
Vaccinated with three doses	2687	711	3398	0	8952	429	9370	11	14,917	282	15,137	62
**Charlson Comorbidity Index**												
0	3013	907	3656	264	52,211	2335	54,179	367	57,564	395	57,906	53
1	314	280	458	136	1944	633	2355	222	1588	118	1676	30
2	156	165	224	97	851	255	1029	77	754	76	810	20
3	112	148	174	86	398	268	535	131	344	64	396	12
**Immunization status**												
No infection, no vaccine	3589	1493	4500	582	42,584	3,050	44,942	692	16,046	176	16,193	29
No infection, 1 dose	0	0	0	0	1,650	129	1,741	38	2,476	26	2,499	3
No infection, 2 doses	0	0	0	0	9,411	244	9,602	53	23,934	141	24,055	20
No infection, 3 doses	0	0	0	0	1,289	55	1,333	11	14,585	276	14,799	62
Infection, no vaccine	6	7	12	1	196	10	203	3	1,302	9	1,311	0
Infection, 1 dose	0	0	0	0	159	2	161	0	917	6	923	0
Infection, 2 doses	0	0	0	0	107	1	108	0	727	14	740	1
Infection, 3 doses	0	0	0	0	8	0	8	0	263	5	268	0

Supplementary Table S3. Absolute numbers of severe diseases and deaths by the period of infection included in the multivariate logistic regression model presented in Table 4. The pre-Omicron period was from September 01, 2020, to December 20, 2021, while the Omicron BA.1 period was from January 01, 2022, to February 28, 2022. Infections occurring before August 31, 2020, were excluded. SD: Standard Deviation.

	Period of infection Sep 01, 2020, to Dec 20, 2021				Period of infection Jan 01, 2022, to Feb 28, 2022			
	Severe disease		Death		Severe disease		Death	
	OR	95%CI	OR	95%CI	OR	95%CI	OR	95%CI
**Immunization status**								
No infection, no vaccine	1		1		1		1	
No infection, 1 dose	0.56	(0.45 - 0.69)	0.47	(0.32 - 0.68)	1.12	(0.72 - 1.73)	1.01	(0.28 - 3.63)
No infection, 2 doses	0.32	(0.28 - 0.37)	0.38	(0.28 - 0.51)	0.38	(0.3 - 0.48)	0.46	(0.25 - 0.86)
No infection, 3 doses	0.23	(0.17 - 0.31)	0.20	(0.11 - 0.36)	0.30	(0.24 - 0.37)	0.20	(0.12 - 0.33)
Infection, no vaccine	0.95	(0.45 - 2.01)	0.97	(0.25 - 3.84)	0.56	(0.28 - 1.12)	-	
Infection, 1 dose	0.31	(0.07 - 1.29)	-		0.46	(0.20 - 1.08)	-	
Infection, 2 doses	0.18	(0.02 - 1.41)	-		0.35	(0.19 - 0.63)	0.06	(0.01 - 0.43)
Infection, 3 doses	-		-		0.16	(0.06 - 0.41)	-	
**Sex**								
Male	1		1		1		1	
Female	0.64	(0.60 - 0.70)	0.5	(0.43 - 0.59)	0.79	(0.67 - 0.93)	0.49	(0.33 - 0.74)
**Age**	1.07	(1.07 - 1.07)	1.14	(1.13 - 1.14)	1.08	(1.07 - 1.08)	1.19	(1.16 - 1.21)
**Charlson Comorbidity Index**								
0	1		1		1		1	
1	1.93	(1.72 - 2.16)	2.33	(1.92 - 2.82)	2.57	(2.03 - 3.24)	2.35	(1.46 - 3.8)
2	1.87	(1.59 - 2.21)	1.86	(1.41 - 2.46)	3.62	(2.73 - 4.79)	3.29	(1.87 - 5.79)
3	3.22	(2.69 - 3.86)	4.93	(3.85 - 6.32)	5.08	(3.70 - 6.96)	2.59	(1.30 - 5.15)

Supplementary material Table S4. Odds ratios of severe disease (i.e., including hospitalization and/or death for COVID-19) and death for COVID-19 by immunization status, adjusted for age, sex, vaccination history, Charlson Comorbidity Index, and SARS-CoV-2 variant, in the pre-Omicron and Omicron BA.1 periods. The pre-Omicron period was from September 01, 2020, to December 20, 2021, while the Omicron BA.1 period was from January 01, 2022, to February 28, 2022. Infections occurring before August 31, 2020, were excluded. OR: Odds Ratio; CI: Confidence interval

	Period of infection Sep 01, 2020, to Dec 20, 2021				Period of infection Jan 01, 2022, to Feb 28, 2022			
	Severe disease		Death		Severe disease		Death	
	Risk (per 1000)	95% CI	Risk (per 1000)	95% CI	Risk (per 1000)	95%CI	Risk (per 1000)	95% CI
**Exposure**								
First infection	59.3	(57.6; 61.0)	13.5	(12.7; 14.4)	11.0	(10.2; 11.8)	2.1	(1.7; 2.4)
Re-infection	49.5	(25.6; 73.5)	10.6	(0.0; 21.9)	7.1	(4.7; 9.5)	0.2	(0.0; 0.5)
Risk difference	-9.8	(-16.8; -2.7)	-2.9	(-6.2; 0.4)	-3.9	(-6.5; -1.2)	-1.9	(-2.4; -1.4)

Supplementary Table S5. Absolute risk and risk difference of severe disease (i.e., including hospitalization and/or death for COVID-19) and of death for COVID-19 for reinfections compared to first infections, adjusted for age, sex, vaccination history, Charlson Comorbidity Index, and SARS-CoV-2 variant. The pre-Omicron period was from September 01, 2020, to December 20, 2021, while the Omicron BA.1 period was from January 01, 2022, to February 28, 2022. Infections occurring before August 31, 2020, were excluded. CI: Confidence interval

	Period of infection Jan 15, 2022 to Feb 28, 2022					
	**Severe disease**			**Death**		
	**OR**	**95%CI**		**OR**	**95%CI**	
**Exposure**						
First infection	1			1		
Re-infection	0.65	0.42	1.02	0.09	0.01	0.67
**Sex**						
Male	1			1		
Female	0.78	0.64	0.95	0.38	0.24	0.61
**Age**	1.08	1.07	1.08	1.17	1.14	1.20
**Vaccination status**						
Unvaccinated	1			1		
Vaccinated with one dose	0.80	0.46	1.40	0.39	0.05	3.11
Vaccinated with two doses	0.44	0.33	0.59	0.48	0.23	1.03
Vaccinated with three doses	0.34	0.26	0.44	0.23	0.13	0.41
**Charlson Comorbidity Index**						
0	1			1		
1	2.48	1.88	3.27	2.15	1.22	3.80
2	3.38	2.42	4.71	3.48	1.86	6.50
3	4.85	3.31	7.10	2.66	1.20	5.86

Supplementary Table S6. Odds ratios of severe disease (i.e., hospitalization and death for COVID-19) and of death for COVID-19 for reinfections compared to first infections, adjusted for age, sex, vaccination history, CCI, and SARS-CoV-2 variant. Only infections occurring between January 15, and February 28, 2022, were included (n=39,830) since all genotyping on samples randomly collected in Reggio Emilia province across this period confirmed the Omicron variant (predominance of BA.1 lineage). OR: Odds Ratio; CI: Confidence interval
